# Audiovisualization of real-time neuroimaging data

**DOI:** 10.1371/journal.pone.0297435

**Published:** 2024-02-21

**Authors:** David N. Thibodeaux, Mohammed A. Shaik, Sharon H. Kim, Venkatakaushik Voleti, Hanzhi T. Zhao, Sam E. Benezra, Chinwendu J. Nwokeabia, Elizabeth M. C. Hillman

**Affiliations:** Laboratory for Functional Optical Imaging, Departments of Biomedical Engineering and Radiology, Mortimer B. Zuckerman Mind Brain Behavior Institute, Columbia University, New York, NY, United States of America; Georgia State University, UNITED STATES

## Abstract

Advancements in brain imaging techniques have significantly expanded the size and complexity of real-time neuroimaging and behavioral data. However, identifying patterns, trends and synchronies within these datasets presents a significant computational challenge. Here, we demonstrate an approach that can translate time-varying neuroimaging data into unique audiovisualizations consisting of audible representations of dynamic data merged with simplified, color-coded movies of spatial components and behavioral recordings. Multiple variables can be encoded as different musical instruments, letting the observer differentiate and track multiple dynamic parameters in parallel. This representation enables intuitive assimilation of these datasets for behavioral correlates and spatiotemporal features such as patterns, rhythms and motifs that could be difficult to detect through conventional data interrogation methods. These audiovisual representations provide a novel perception of the organization and patterns of real-time activity in the brain, and offer an intuitive and compelling method for complex data visualization for a wider range of applications.

## Introduction

Current techniques for in vivo brain imaging, such as functional magnetic resonance imaging (fMRI), wide field optical mapping (WFOM), 2-photon and light-sheet microscopy provide large quantities of multi-dimensional, dynamic data. Recent improvements to these techniques allow real-time recordings, enabling observation of spontaneous events, as well as compelling ‘resting state’ activity [1–6]. This inundation of data to analyze and understand brings with it a challenging task: to take large datasets, and distill them into concise representations that preserve the information content of the data and offer insights into the mechanisms generating the spatiotemporal patterns observed [7, 8]. Dimensionality reduction, or spatiotemporal unimixing is becoming mainstream in the analysis of in-vivo microscopy data to extract the shapes and time-courses of firing neurons [9]. This approach is similarly the basis of resting state fMRI analysis, which extracts spatial ‘functional connectivity networks’ based on the temporal correlations of different regions of the brain [10]. However, the outputs of these spatiotemporal unmixing methods are rarely re-combined into meaningful representations from which real-time interactions and inter-component spatiotemporal patterns and dynamics can be easily appreciated.

Modern neuroscience experiments also include recordings of behavior, such as whisking, and spontaneous running, stimulus presentations, tasks such as lever pushing, and parameters such as task performance speed and correctness. These recordings often begin as video streams. Feature extraction from these behavioral recordings is achievable [11, 12], and can provide an input for machine learning algorithms to generate predictive models. However, it can be challenging to determine which of the many features in the video are relevant, and spatial tracking parameters extracted from videos will not necessarily be linearly related to neural representations (e.g. breathing rate, or the speed of a movement). Conversely, the human brain is very good at such feature extraction from video streams. The problem is that comparing two or more video representations (brain imaging data and behavioral recordings) at the same time is very challenging for the human visual system to achieve.

To overcome these issues, we demonstrate here methods for representation of brain imaging data in both the visible and audible space, providing an intuitive representation of high-dimensional data that can be listened to in parallel with viewing videos of behaviors, and spatial representations of the data. Audible representations of electrophysiology signals have long been used to guide electrode placement and distinguish signal from noise, while enabling simultaneous use of the eyes and hands. The idea of representing EEG and fMRI data from awake humans as audio streams has also been demonstrated previously [13, 14], in addition to data from mouse brain slices [15]. Our approach improves upon these demonstrations by providing a toolkit for routine encoding of a wide variety of parameters by leveraging sound’s ability to simultaneously depict multiple dimensions of dynamic information in parallel. Pitch, volume, note velocity, attack speed, stereo sound and even musical instrument type are all parameters that can all be leveraged for auditory stream encoding. Since all of these aspects are easily recognized and unmixed by the human auditory system [16], a great deal of information can be compressed into a single audio stream for real-time evaluation of brain imaging data and associated behavior, providing a unique way to recognize patterns, motifs, co-activations, delays, rhythms and repetitions not easily noticed by eye alone. As a further dimension for encoding, our approach also provides visual representations of spatiotemporal dynamics of the data using colors, further expanding our ability to utilize our sensory system to integrate and interpret the properties of each dynamic system.

We demonstrate our approach on three different types of experimental neuroimaging data with audiovisualization of gradually increasing complexity: Wide-field optical mapping (WFOM) of neural activity to compare the mouse brain under awake and anesthetized conditions (ketamine/xylazine), cellular-level resolution recordings of apical dendrite intracellular calcium activity in the awake mouse brain using swept confocally aligned planar excitation (SCAPE) microscopy [1], and wide-field simultaneous neural and hemodynamic recordings of the awake mouse cortex, along with behavioral recordings. We provide our python-based graphical user interface PyAnthem (Automated Neuroimaging Timecourse Heuristic Methodology) that is capable of reproducing these audiovisualizations and extending the use of this method to wider ranges of dynamic spatiotemporal data. See S1 Appendix for full details of this new open-source toolkit.

## Materials and methods

Please note that all in-vivo brain imaging datasets shown here were collected as parts of different scientific studies into brain dynamics, neurovascular coupling and anesthesia effects, and not for the sole purpose of this audiovisualization project [3, 5, 6, 17–19].

### Wide Field Optical Mapping (WFOM) data collection (experiments 1 and 3)

#### WFOM imaging data collection

WFOM is an in-vivo optical imaging technique capable of recording real-time neuronal activity and hemodynamics across the dorsal cortex of awake, behaving mice. We leverage transgenic mice expressing calcium-sensitive green fluorescent protein (GCaMP) in excitatory neurons, which can provide an ensemble spatiotemporal image of neuronal activity when excited with blue light from a light emitting diode (LED) illumination through a thinned skull cranial window. Reflectance measurements using green and red LEDs can be spectroscopically converted to dynamic maps of oxy-, deoxy- and total hemoglobin, signals which can also be used to remove the effects of absorption cross-talk on neuronal fluorescence signals.

During awake WFOM imaging experiments, mice are head-fixed and positioned on a low-resistance custom-made horizontal wheel that enables them to move and walk, and imaged for up to 90 minutes per session. The mouse’s behavior is recorded using synchronous webcam cameras capturing body and pupil movements. Specific details for each experimental dataset are provided in the Results section.

#### WFOM imaging system details

Our custom-built WFOM system consists of three high powered LEDs (Thorlabs, M490L2, M535L2 and M625L3) at wavelengths of 490 nm (Blue), 535 nm (Green), 625 nm (Red), that are strobed to capture hemodynamic (red and green) and neural (blue) fluctuations simultaneously. Synchronous images are captured using an Andor Zyla sCMOS camera running at 31.22 Hz and an exposure time of 23.4 ms (resulting in an imaging frame rate of 10.4 Hz for each variable). A long-pass filter mounted in front of the camera blocks blue excitation light (FF01-496/LP-25, Semrock) in addition to an infrared shortpass filter to block behavioral monitoring light. Band-pass filters are placed in front of each LED (FF01-475/28 for blue, FF01-530/43 for green, and FF01-623/24 for red). For hemodynamic correction, red and green reflectance measurements are used to derive estimated values of hemodynamic absorption contributions to (blue) excitation and (green) fluorescence emission light as described previously [2]. The mouse’s behavior is recorded during WFOM imaging using two independent webcams (PS3 Eye), with a filtered infrared LED light source illuminating the mouse and a matching infrared filter on the webcams to prevent cross-talk with imaging light.

#### Animal preparation

All animal procedures were reviewed and approved by the Institutional Animal Care and Use Committee at Columbia University (protocol AC-AAAS3453). For all WFOM imaging experiments, we used adult C57BL/6J-Tg(Thy1-GCaMP6f) mice (purchased from Jackson Labs and bred in-house). Mice were initially anesthetized with isoflurane and underwent a thinned-skull craniotomy over the cortex between coronal and lambdoid sutures, and implanted with a laser-cut acrylic head plate for restraint. The thinned-skull craniotomy was then protected by an optically clear cyanoacrylate layer (applied during surgery) to improve transparency and reduce bone regrowth. After surgery, all mice underwent a two-day post-operative recovery period with analgesia before imaging began.

### SCAPE microscopy data collection (experiment 2)

#### SCAPE imaging data collection

SCAPE microscopy is a single-objective high-speed 3D volumetric light sheet imaging technique capable of imaging cellular-level GCaMP activity in awake behaving mice at both high spatial and temporal resolution [1, 20] (**Fig 3A and 3B**). A mouse was prepared using viral transfection to produce sparse GCaMP6f labeling in apical dendrites of layer 5 neurons, and SCAPE data was acquired through a glass cranial window while the mouse was head-fixed but awake. SCAPE datasets were acquired with 488 nm (blue) excitation was acquired for a 60-second trial at 9.4 volumes per second. The size of the imaging volume was 374 x 1032 x 174 microns with a voxel size of 2.5 x 1.27 x 1.17 microns (x-y-z) [1, 18, 20].

#### Animal preparation

Adult wild-type C57BL mice were initially anesthetized with isoflurane and underwent viral injection (AAV9.Syn.GCaMP6f) into the barrel cortex to label layer 5 cortical neurons with the calcium sensitive fluorescent protein GCaMP6f. Mice were then implanted with a glass cranial window (dura removed) and a head-fixation head plate and allowed to recover with analgesia. After several weeks, apical dendrites reaching up to cortical layers 1–3 were sparsely expressing the fluorescent calcium indicator and could be recorded using SCAPE microscopy.

## Results

### Basic audiovisualization workflow

**Fig 1:**
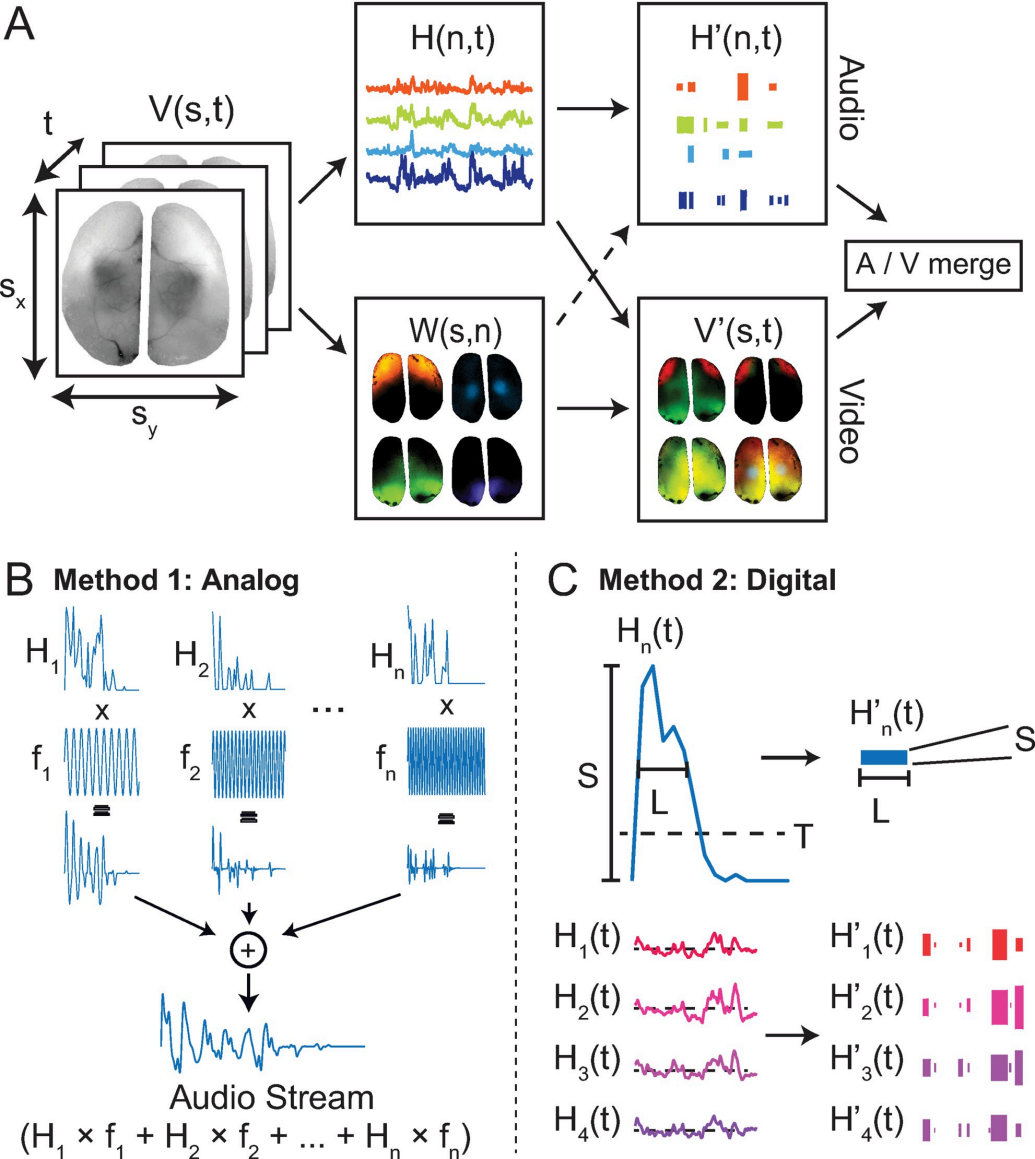
The audiovisualization process for a generalized dataset. A) The dataset V(s,t) is represented as n temporal (H(n,t)) and spatial (W(s,n)) components, which are then used to create both an audible representation and a color-based remixed visualization of the dataset respectively. Datasets can be 3D or 4D, but a 3D WFOM dataset is used for this illustration. B) Audio generation method 1 (analog). Basis timecourses modulate static tones (either sinusoids or software instrument sounds), and are played simultaneously, producing an audio stream. C) Audio generation method 2 (digital). Basis timecourses are converted to MIDI note events that can be rendered as musical instruments. Here, each event is defined as a portion of the timecourses that exceeds threshold T, with length L. The strength of the note S is the peak amplitude of the note within the window L.

The 4 steps of data processing for audiovisualization are shown in **Fig 1**. First, data must be spatiotemporally unmixed into a dimensionality-reduced representation. This step is best achieved via the analysis method most common to the imaging modality being used, but could include principal component analysis (PCA), non-negative matrix factorization, k-means clustering, seed-based non-negative least squares fitting or other specialized blind source separation methods [21–23]. The goal is to represent the data as a linear combination of n temporal (H(n,t)) and spatial (W(s,n)) components assuming that the data can be represented as:

V(s,t)≈W(s,n)⋅H(n,t) (1)

Videos are then generated by combining W(s,n) and H(n,t) using color-based remixing based on an n x 3 color map C. At each time-point T, the image corresponds to:

V’(s,T,1:3)=W(s,n)⋅diag(H(n,T))⋅C(n,1:3). (2)

Audio-streams are generated from the temporal components H(n,t) either by directly modulating pure waveforms based on signal amplitude (‘analog’ method, **Fig 1B**), or by converting time-courses to MIDI messages for increased control over note parametrization (‘digital’ method, **Fig 1C**). The pitch of the note assigned to each component n can be chosen based on some property of the data, such as the spatial position of each component in W(s,n). The final step is to merge synchronized audio and video streams into a combined movie. All of these basic functions can be performed within our open-source PyAnthem software package as described further in S1 Appendix. A summary of the analysis and preprocessing techniques for each example shown below is detailed in **Table 1**.

**Table 1:** Summary of experimental analysis steps used to create audiovisualizations.

Experiment	Method	Data Sources	Audio conversion technique	Time course selection	Number of components
**1**	WFOM	Neural (GCaMP6f)	Analog	K-means	18
**2**	SCAPE	Neural (GCaMP6f)	Digital	K-means	43
**3**	WFOM	Neural (GCaMP6f), Hemodynamic, Behavior	Analog & Digital	K-means	12

### Experiment 1 –Neural activity in the mouse cortex, awake vs ketamine/xylazine anesthesia

**Fig 2:**
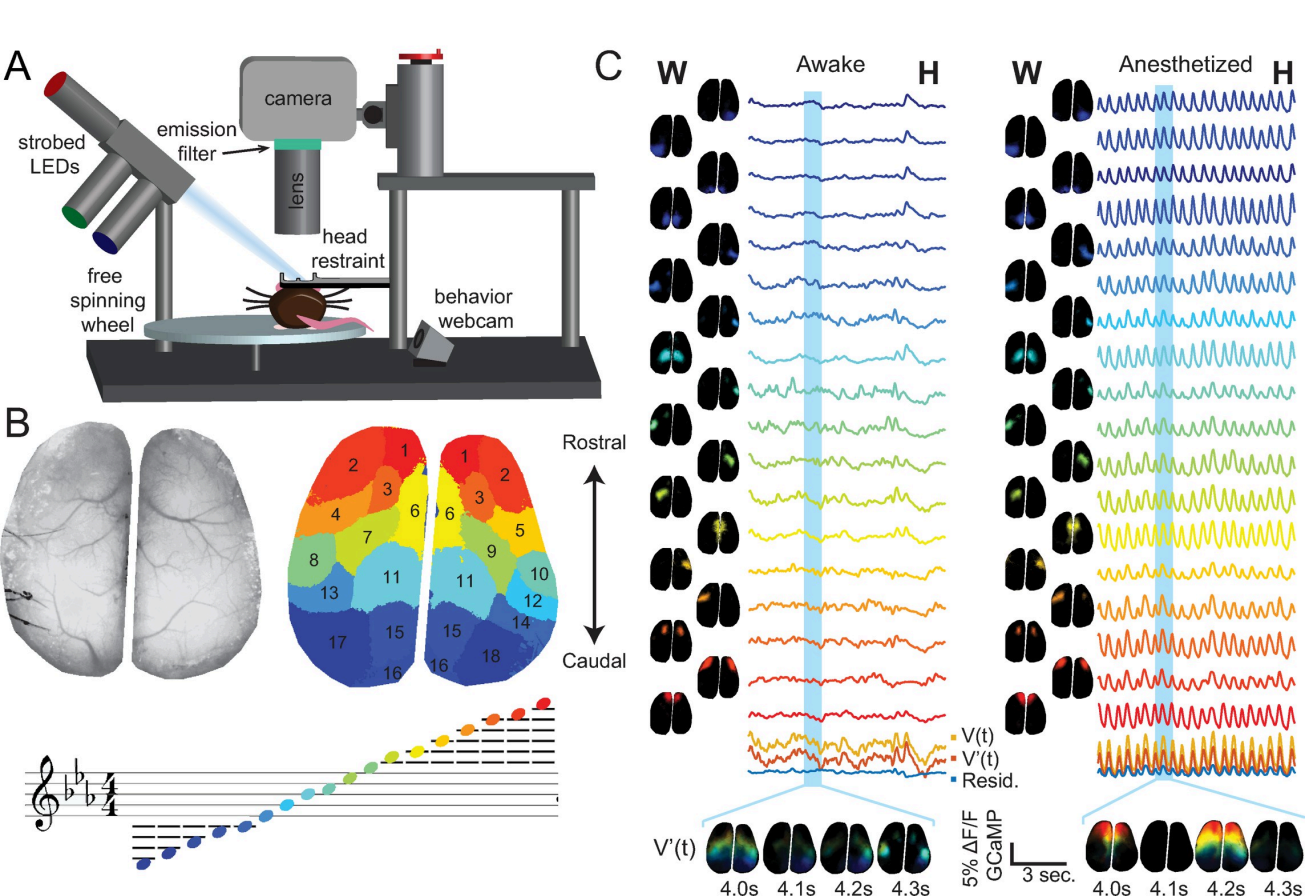
Simple audiovisualization of wide field neural activity in awake vs. anesthetized mouse. A) Schematic of WFOM setup. B) Wide field image showing dorsal cortex of the thinned-skull mouse (left), k-means clustering output of neural activity (right), and note assignment for each k-means cluster (bottom). This k-means output was used to obtain the basis timecourses for both awake and anesthetized datasets. C) Timecourses obtained from ROIs defined by clustering in B are used to derive spatial components using Nonnegative Least Squares (NNLS) fitting to each pixel’s timecourse. The basis timecourses are then used to create an audio stream. Plots at the bottom show the average original-data timeseries (V(t), yellow) compared to the linear model (V’(t), orange), and the residual V(t)-V’(t) is plotted below it in blue. (see **S1 and S2 Movies** for final audiovisualizations).

As a simple example, we start with audiovisualization of a dynamic 2D WFOM dataset in which we spatiotemporally unmix and then represent data as time-varying pitches and color-coded components. Dynamic images of neural activity over the dorsal surface of the living mouse brain were acquired using WFOM through thinned skull in a Thy1-GCaMP6f mouse [3] (**Fig 2A**). Data was pre-processed to remove hemodynamic contamination of GCaMP fluorescence [2] (detailed in Materials and Methods).

This dataset was acquired as part of a study to explore the effects of anesthetics on resting state neural activity in the mouse brain. The mouse was initially imaged awake and head-fixed on a freely moving wheel to record awake resting state data. The animal was then removed from the imaging rig and an anesthetic dose of Ketamine/xylazine (115 mg/kg Ketamine, 11.5 mg/kg Xylazine) was injected intraperitoneally. After the animal was fully anesthetized, it was again head-fixed and imaging data was acquired.

#### Dimensionality reduction

To estimate the primary temporal components of the data in an unsupervised manner, bilateral GCaMP data from the awake experiment was k-means clustered into 18 components via correlation distance measure. These clusters were then ordered according to their centroid position from the front to the back of the brain, and each component was assigned colors from the Matlab™ ‘jet’ color map (**Fig 2B**). We ensured that input data for this clustering corresponded to periods when the mouse was not running. Eighteen basis timecourses H(n,t) were then obtained using eroded k-means spatial clusters as regions of interest (ROIs). Non-Negative Least Squares (NNLS) fitting was then used to generate 18 spatial maps corresponding to the weight of each time-course W(s,n) in each pixel of the raw dataset V(s,t) [24]. Re-multiplying W and H yields model data V’(s,t) that can be compared to the original data to observe information that was lost in the dimensionality-reduced linear approximation (e.g. see **S1 and S2 Movies**). The same eroded k-means ROIs were used to extract basis time-courses from the ketamine/xylazine-anesthetized dataset, and then NNLS was used to generate that dataset’s corresponding spatial components and linear representation.

#### Video stream generation

Using the color assignments shown in **Fig 2B**, spatial components W and temporal components H were multiplied and remixed to create dynamic color visualizations of neural activity. To clearly show periods of increased neural activity, all values below zero are represented as black in movies, so careful selection of this baseline is needed if significant decreases are present (here, the baseline chosen was the mean of the interval used to calculate GCaMP %ΔF/F the k-means clusters, i.e. at rest).

#### Analog audio stream generation + pitch encodes position

H(n,t) was used to create an audio stream by multiplying each temporal component with a unique audio frequency sinusoid and then summing all of the components together (**Fig 1B**). The note pitches chosen were an ascending Cmin^7^ chord (C, E♭, G, and B♭, spanning 5 octaves across 18 components), ordered according to the centroid position of the W components ascending from back to front of the brain. **Fig 2B** shows the ordering of the 18 components and the corresponding musical note assigned to each. We note that it is important not to simply use arbitrary integer values for note frequencies, and to instead use natural note frequencies from the chromatic 12-tone scale to improve listenability. Finally, the audio stream was added as a soundtrack to the neural data spatial component video V’(s,t,c).

#### Effects of anesthesia on brain dynamics

**S1 and S2 Movies** show the outputs from the above analysis on a mouse before and after induction of ketamine/xylazine anesthesia respectively. As can be appreciated from these representations, the awake brain exhibits a variety of different activation patterns, typical of resting state datasets previously reported [2], whereas ketamine/xylazine anesthesia resulted in a dramatic, repetitive rostro caudal wave of neural activity. This pattern is consistent with prior reports of slow wave neural dynamics under ketamine [25–27]. Audiovisual representations of this activity reveal a consistent rhythmic pattern from high to low notes, and yet it is also possible to perceive that each wave is not completely unidirectional, as some waves retrace forwards or originate in peripheral components.

### Experiment 2 ‐ SCAPE microscopy of apical dendrites of layer 5 neurons in awake mouse brain

Audiovisualization can also be applied to microscopy data. In the following example, we utilize real-time 3D SCAPE microscopy data capturing spontaneous calcium events in apical dendrites of neurons in the awake mouse somatosensory cortex. By converting neuronal events to a midi format, we enable use of a piano VST (Virtual Software Technology). We use timing features of the data to choose note assignments.

#### Dimensionality reduction

After background subtraction and detrending of the 4-dimensional SCAPE dataset, each voxel’s time series V(s_x_,s_y_,s_z_,t_1-n_) was checked for values that exceeded a z-score of 4. If more than 5% of the values in a voxel exceeded this threshold over the full time-series it was passed to the next clustering step, otherwise it was removed. The remaining voxels were then clustered into 85 components using K-means clustering. The resulting basis time courses were then used to create spatial maps using NNLS. These 85 components were then manually pruned down to 43 components by removing those that did not show well-defined morphologies in their spatial maps. Each 3D spatial component was median filtered (3x3x3 voxel filter) spatially to reduce background.

**Fig 3:**
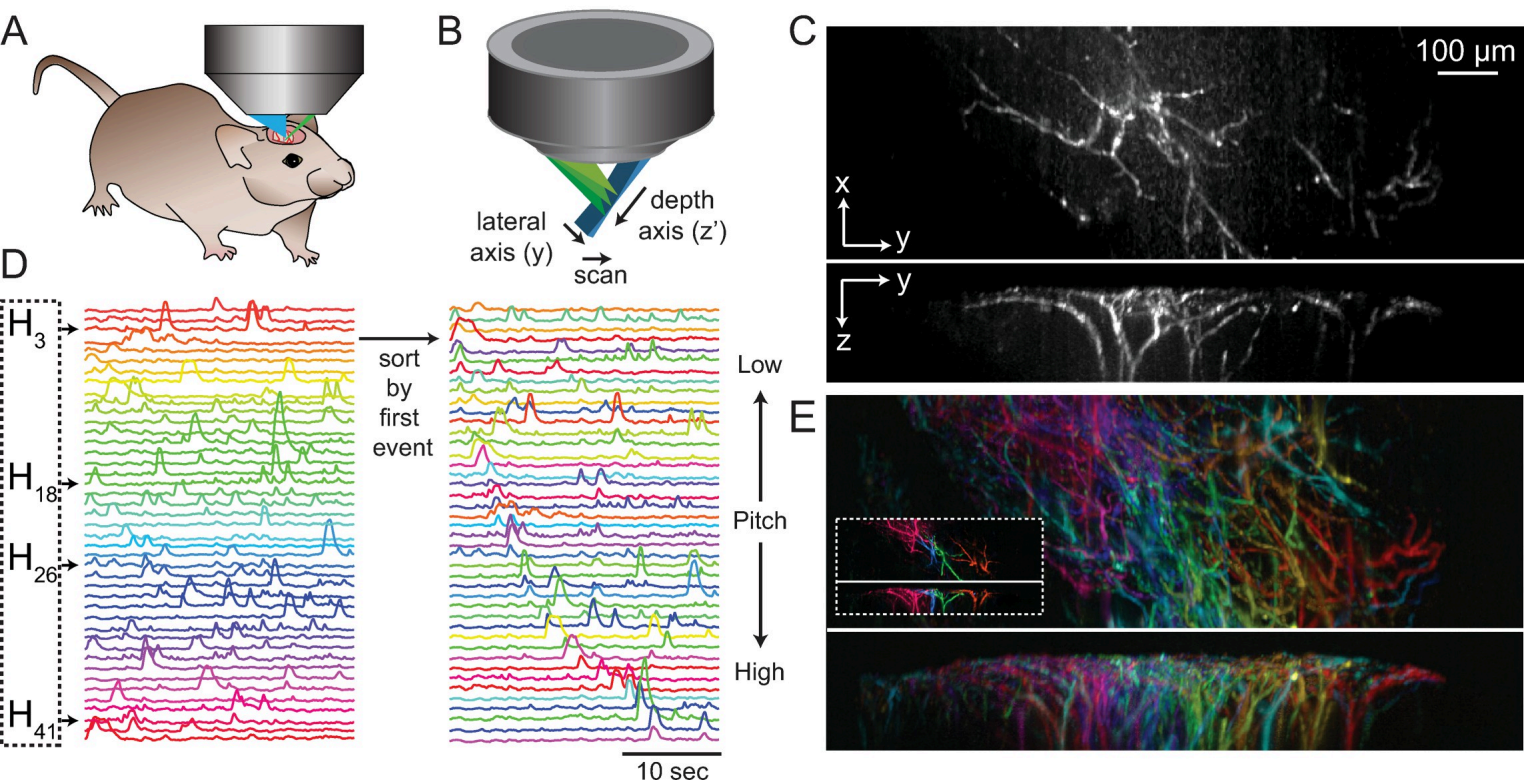
Audiovisualization of a 4D SCAPE microscopy dataset. A-B) Diagrams of SCAPE objective and mouse placement. C) Maximum Intensity Projections (MIP), top and side view of one time-point in the raw dataset during a dendritic firing event. D) Basis timecourses (H), arranged spatially on the left, and then arranged in order of first spike, with the original spatial color assignments kept. Audible note frequency follows the spike ordered components on the right, rather than the spatially arranged components on the left. E) MIP of top and side projection of all 43 spatial components after unmixing, color coded spatially from right (red) to left (pink). Inset shows four example components, which are also highlighted below in D. See **S2 Fig** for individual images of all spatial components. See **S3 Movie** for dynamic audiovisualization.

The resulting 43 spatial (W) and temporal (H) components are shown in **Fig 3C**. In this case, the unmixed spatial components W(i,j,k,n) are 3-dimensional in space. Components were color-coded according to the component’s centroid in the lateral dimension. **S3 Movie** (S3 Movie) shows results as 3-view maximum intensity projections (MIPs).

#### Digital audio stream generation using MIDI encoding + pitch encodes event sequence

To improve both the ease of listening and clarity of the audio stream compared to experiment 1, instead of simply summing sinusoids and varying their volume, here we encoded neural activity as a piano note. The percussive nature of a piano strike (fast onset followed by slow decay) allows the listener to easily hear the complexity of multiple event onsets while still perceiving the duration of each event.

To convert extracted time-courses H(n,t) into musical notes, distinct neural events need to be identified and represented by their magnitude, time of onset and duration of activity. For microscopy data in which events clearly emerge from a dark background, event identification is possible with a simple threshold, although care should be taken not to set the threshold too low, to avoid grouping multiple events into one sustained note. All events are then represented based on their peak amplitude, onset and duration (**Fig 3C**), such that small, weaker events are present but audibly scaled to match their magnitude. This digitization can then easily be converted into the MIDI message format in which each note is assigned a; note on (sec), note off (sec), note strength (ranging from 0 to 127), chromatic key choice (0 to 127, with 60 as middle C, or C3), and instrument (here, we used piano). This format can be converted into music using any VST, including Garage Band or REAPER, and is performed automatically in PyAnthem (see **S1 Appendix**).

In this example, rather than using note pitch to encode spatial position (as in experiment 1), we chose to sort components by the timing of their first event (**Fig 3D**). This means that that notes increase in pitch over time, but will gradually become more mixed as the same neuron fires a second, third or fourth time during the trial.

#### Audiovisualization of SCAPE microscopy of mouse dendritic activity

The resulting audiovisualization is shown in **S3 Movie**. Ordering notes according to event timing gives a unique perspective on the relative timing and frequency of firing events of each neuron. We note that labeling of neurons in this case was sparse, and therefore does not represent the full activity of the somatosensory region. However, this representation permits assessment of the firing properties of single neurons that could be readily compared to real-time behavioral recordings, presentation of stimuli or the performance of tasks.

We also note that this analysis sought a deliberately low-dimensional representation of this data, which clustered similar time-courses together to yield groups of well-correlated pixels that belong to a dendritic tree, and differ sufficiently from other pixels over time to differentiate them from another dendritic tree (see **S2 Fig** for individual components). This low dimensionality removed higher order spatiotemporal noise in the dataset, but also did not seek to discover subtler differences in the timing of activity patterns along each dendrite or that may have differed within a dendritic tree from one event to the next.

### Experiment 3 ‐ Neural and hemodynamic recordings in the awake, behaving mouse

Here we demonstrate a full pipeline in which WFOM data of both neural activity and hemodynamics across the dorsal surface of an awake mouse are represented simultaneously by two different musical instruments, rendered in parallel with a video stream of behavioral recordings.

#### Dimensionality reduction and video stream generation

**Fig 4:**
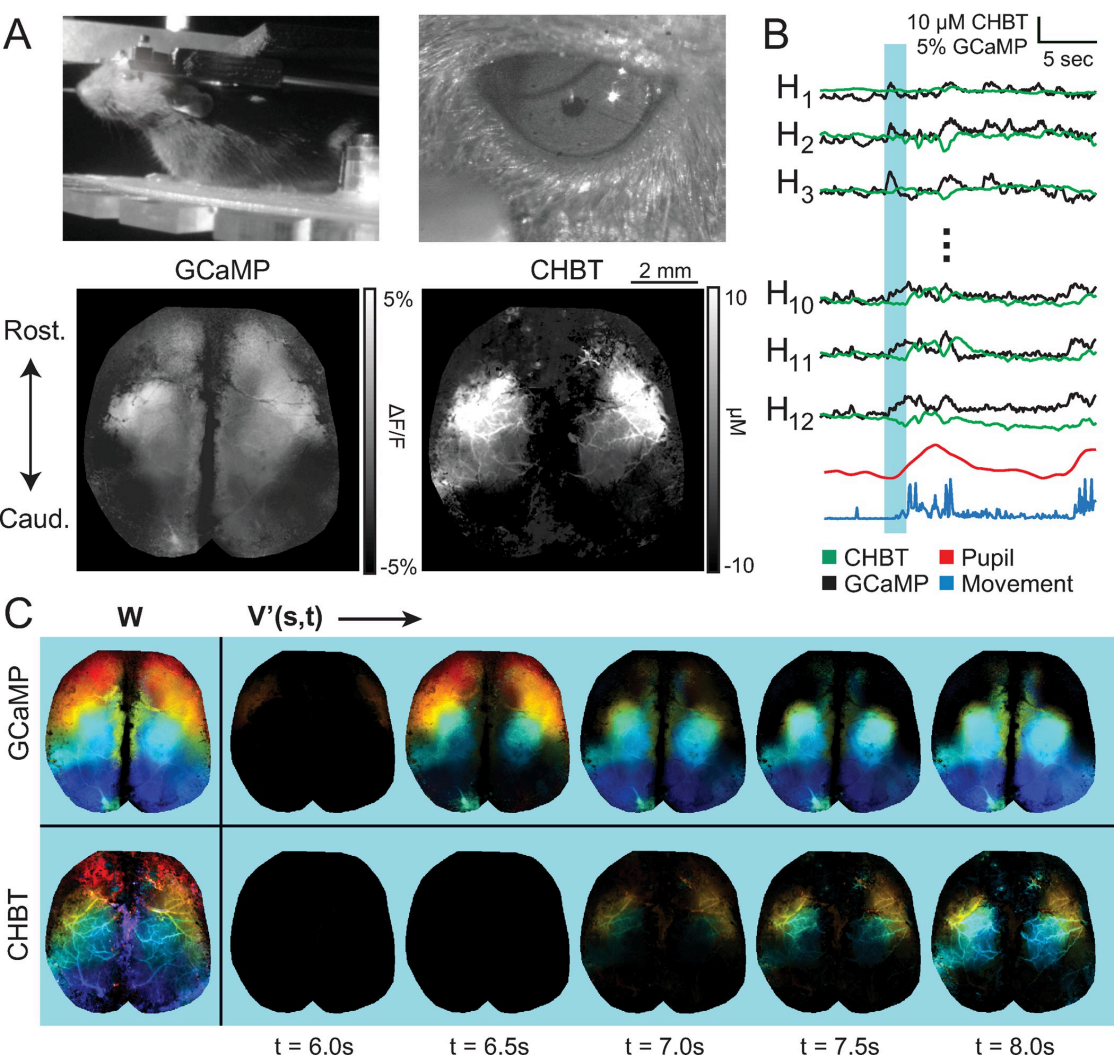
Audiovisualization of both neural and hemodynamic WFOM data with simultaneous behavior in an awake mouse. A) Example frames of behavioral, pupil, GCaMP and hemodynamic data. B) Plot of temporal basis timecourses, extracted using k-means clustering. C) Color mixed representation of NNLS output W, and reconstructed color mixed dataset at example timepoints from both neural (top) and hemodynamic (bottom) datastreams. The blue bar in B represents the time period of example data shown here. See **S4 Movie** for full audiovisualization.

The same approach as in experiment 1 was used to reduce the collected neural data into 12 components (k-means clustering, followed by NNLS to extract spatial maps W). The same eroded ROIs, defined by the neural data were then also used to obtain basis timecourses for the hemodynamic data, followed by NNLS to extract the hemodynamic W. Color remixing was used to create movies of both hemodynamic and neural datasets in the same way as experiment 1. Colors were chosen from a jet color map, and assigned from the front (red) to the back (blue) of the dorsal surface of the brain (see **Fig 4C**) in an identical way for both neural and hemodynamic components. A composite movie was generated that includes behavioral and pupil data, after temporal synchronization between all of the video streams.

#### Combined analog and digital audio stream generation + instrument encodes measurable

The audio stream was generated using the analog method (**Fig 1B**) for hemodynamic activity and the digital method (**Fig 1C**) for neural activity. The neural data was encoded as piano notes, while the slower, more continuous nature of hemodynamic fluctuations were encoded as violin. These two instruments provide a clear audible difference in the two dynamic datasets, despite their simultaneity. A Cmin^7^ chord was used for both data streams, with notes ascending from the back (rostral) to the front (caudal) of the brain. It should be noted that all audible sounds represented positive signals, which are dependent on the chosen baseline. Thus, it was important for this audiovisualization to select a baseline that was during a relatively quiet period of activity to avoid losing sub-threshold events.

#### Audiovisualization of neurovascular dynamics during spontaneous behavior

**S4 Movie** (S4 Movie) shows four simultaneous video streams–behavioral, pupil, neural and hemodynamic measures of the awake behaving mouse. First, it is easily observed that sharp increases in neural activity, represented by the piano notes, are typically followed by slower matching hemodynamic chords, consistent with the properties of neurovascular coupling in the brain acting as a delayed spatiotemporal low pass filter of neural activity [28]. The behavior of the mouse is also clearly depicted in the audiovisualization of brain data. Onsets of running are striking events including significant activation of hindpaw regions, with hemodynamics of the region following behind. However, differences in the sequence of neural activation for different running bouts can also be discerned. At 15 seconds, the mouse begins grooming, and these finer movements are well represented by short, quiet notes that represent activation of forepaw somatosensory and motor regions. Activation of the bilateral visual cortex is seen at the start of the run when the WFOM illumination LEDs first turn on (see **S1 Fig** for an anatomical map of functional regions of the mouse cortex).

Choosing a suitable baseline and thresholds is particularly important for audiovisualization of datasets that are more smoothly varying over time. If a chosen baseline is too high or low, important audio events may not reach threshold, or may be saturated and difficult to distinguish from one another. The baseline of basis time-courses should always be inspected, and should generally be chosen from a period of relative quiescence. Data can also be filtered or detrended (as in experiment 2) or represented on a non-linear or logarithmic scale to emphasize specific data differentials where appropriate. Where signal decreases below baseline are of particular interest, they could be represented as an additional sound or instrument, and could be represented as grayscale or an additional color in spatial representations.

## Discussion

The application of audiovisualization to real-time neuroimaging data was demonstrated for a variety of different applications. We showed that both mesoscale and cellular-level microscopy recordings of brain activity can be assimilated, with a different perspective offered by reducing large and complex datasets to simple, easily accessible audio streams coupled with relevant visual representations of that activity and / or simultaneously acquired variables such as behavior. Although we do not suggest that audiovisualization be the first step to screen through banks of large data, we have found this technique to be valuable to gain perceptions of complex spatiotemporal features of data in different conditions. We use this information to guide subsequent quantitative extraction of features for hypothesis testing relating to rhythms, motifs, abnormal activity and behavioral representations in both neural and hemodynamic data.

While machine learning and AI are increasingly enabling screening of large datasets for patterns and correlates, initialization of these algorithms and interpretation of their outputs can be challenging [29, 30]. The human auditory and visual systems are incredibly sophisticated, and are able to hear and see patterns and features that exceed the current ability of computers. Our senses can remember, integrate over time, detect patterns, selectively amplify and focus on features independent of their amplitude, and interpret parallel streams of multisensory information. Audiovisualization of data leverages this human ability to enable assimilation of many variables in parallel by merging diverse experimental variables into interpretable representations that fill a much larger portion of our sensory space than classical observations of grayscale data.

This audiovisualization approach can be applied to a variety of dynamic data streams including fMRI, other functional microscopy datasets, and even far beyond analysis of just brain activity. We note that additional perception can be achieved if data is looped, sped up or slowed down to different degrees, depending on the data type. Aspects of animal behavior such as movement, pupil size, heart rate, and other vital signals can also be incorporated into audiovisualization depending on the needs of the application. For example, pupil area and movement (shown in **Fig 4B**) could also be included as an audible signal, in addition to other behavioral measures, such as heart rate or fine motor movements. Percussion instruments or other discernable types of sound could also be incorporated.

One further aspect of this work is the demonstration that real-time neural and hemodynamic activity in the brain actually has similar patterns and rhythms to composed music. We intuit that this music-like nature of whole-brain activity could perhaps relate to the timing patterns of our own brain’s activity and perhaps our brain’s state-dependent preferences for different rhythms and patterns of music.

## Supporting information

S1 Appendix The PyAnthem graphical user interface. Description, details and usage instructions for shared audiovisualization software.
(PDF)

S1 Fig Color-coded cortical functional area atlas for a similar view to that captured in WFOM data. Adapted from the Allen Institute Brain Atlas.
(PDF)

S2 Fig All spatial components of dendrites extracted from 4D SCAPE microscopy dataset (experiment 2). Each panel shows top and side maximum intensity projections (MIPs) of spatial (W) components.
(PDF)

S1 Movie Audiovisualization of neural activity from the dorsal surface of the thinned skull cortex of the awake mouse. Left: Raw GCaMP activity. Middle: Spatiotemporally unmixed linear model, created by multiplying temporal H (obtained by k-means clustering into 18 ROIs) with spatial W (obtained as an output of NNLS, where H was used as the input). Right: color remixed reconstructed model data, where each component of W was assigned a unique color from the jet color map, arranged from top (red) to bottom (blue). Movie’s soundtrack uses analog (sine-wave) based audio encoding of temporal patterns of each spatial component, ordered in an ascending Cmin^7^ scale from the back (bottom) to the front (top) of the brain. *Note*: *Movie has sound*.
(MP4)

S2 Movie Audiovisualization of neural activity from the dorsal surface of the thinned skull cortex of the ketamine/xylazine anesthetized mouse. Left: Raw GCaMP activity. Middle: Reconstructed data, created by multiplying temporal H (obtained by k-means clustering into 18 ROIs) with spatial W (obtained as an output of NNLS, where H was used as the input). Right: color remixed reconstructed model data, where each component of W was assigned a unique color from the jet color map, arranged from top (red) to bottom (blue). Movie’s soundtrack uses analog (sine-wave) based audio encoding of temporal patterns of each spatial component, ordered in an ascending Cmin^7^ scale from the back (bottom) to the front (top) of the brain. *Note*: *Movie has sound*.
(MP4)

S3 Movie Audiovisualization of SCAPE microscopy data capturing calcium activity in apical dendrites in the awake mouse brain. Panels show top and side maximum intensity projections of color-encoded re-mixed spatial components. Dimensionality reduction was applied to voxels in which at least 5% of values over time exceeded a z-score of 4. Time-course from these voxels were then k-means clustered, and the resulting timecourses were used as an input for NNLS. 43 output components were color-coded from left to right using an HSV color map. Movie’s soundtrack depicts supra-threshold events as piano notes and were chosen on an ascending scale according to order of activity. *Note*: *Movie has sound*.
(MP4)

S4 Movie Audiovisualization of neural activity and blood flow from the dorsal surface of the thinned skull cortex of the awake mouse. Behavioral data (top), neuronal activity (GCaMP6f) (bottom left), and cortical hemodynamics (bottom right). Webcam data was acquired simultaneously using two PS3 Eye webcams. Raw GCaMP data was k-means clustered to derive regions of interest (ROIs) from which to extract 12 basis time-courses from both neural and hemodynamic data-streams. Corresponding spatial components fitting a linear model to the original data were derived using non-negative least-squares fitting. Spatial components were then color-coded and re-combined for both datasets, with colors from the Matlab™ jet color map ordered from the front (top, red) to the back (bottom, blue) of the brain. Time-courses for each ROI were converted into audible representations, combined in the movie’s soundtrack as piano notes for neural activity and violin as hemodynamics. *Note*: *Movie has sound*.
(MP4)

S1 File (PDF)
