# Supplementary material for: Audiovisualization of real-time neuroimaging data
Source: PLoS One. 2024 Feb 21;19(2):e0297435. doi: 10.1371/journal.pone.0297435 (PMC10881001; doi:10.1371/journal.pone.0297435)
Supplement: S1 Appendix — Description, details and usage instructions for shared audiovisualization software. (PDF) [file pone.0297435.s001.pdf]

The PyAnthem Graphical User Interface

We have developed an open-source, Python based graphical user interface (GUI) called PyAnthem which enables conversion of dynamic datasets into audiovisualizations using the same techniques described in this manuscript. Full instructions, along with the installation guide below can be found at https://github.com/nicthib/PyAnthem. This tool has the following features:

1. Converts image time-series datasets to visual and audible representations.
2. Allows adjustment of a variety of video and audio parameters.
3. Performs in-place video and audio merge.
4. Provides both GUI and Command-line interfaces.
5. Includes example datasets to get started.
6. Provides easy installation with an open-source Anaconda environment.

Overview

The GUI accepts data that has already been spatiotemporally unmixed, although we also provide basic code to implement dimensionality reduction of a range of data types, first by k-means clustering the data to obtain temporal ROIs H, and then by performing NNLS to obtain spatial components W. The output of the GUI is a fully merged movie with audio soundtrack, although the package can also output renderings at intermediate steps, such as the visualization, or MIDI format outputs for use in external audio programs. **S1 Figure 1** shows a screen-shot of the GUI and basic functionality is detailed briefly below. All GUI components are referred to using ***bold text*** below.

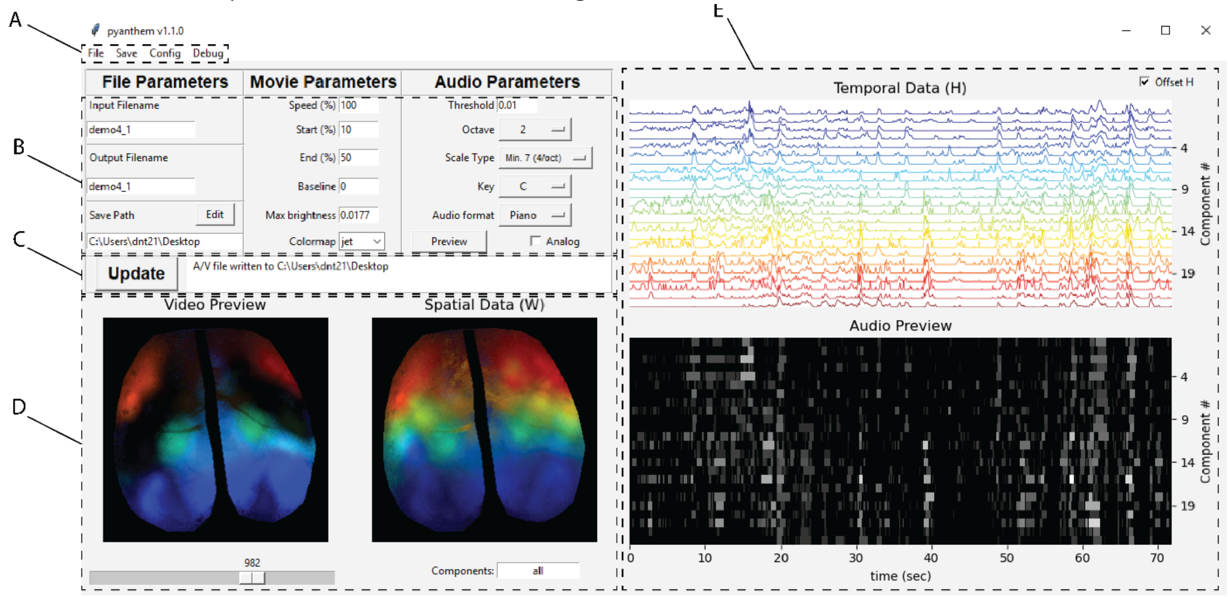

S1 Figure 1 - PyAnthem Graphical User Interface for creating audiovisualizations. Data is imported in the .mat or .h5 format using the file dialog in Box A, and should contain a temporal variable (n x t) and a spatial variable (s_x x s_y x n). Parameters for the audio and video outputs are adjusted in Box B. Preview updates and GUI status are shown in Box C. A video preview and illustration of the spatial components are shown in Box D. Raw temporal data and an audio preview are shown in Box E. Finally, files can be saved using the Save menu in Box A.

Data format

Pyanthem was primarily developed to interpret matrix-decomposed functional imaging datasets - for example, a dataset **V** with shape `[height,width,time]`, is decomposed into two matrixes: **W** with shape `[height*width,n]`, and **H** with shape `[n,time]` such that `H x W = V`. Here, n represents the number of variables represented by the decomposition. There are various techniques used to decompose matrixes - two popular techniques include Non-negative Matrix Factorization (NMF), and Singular Value Decomposition (SVD).

Here's a visual illustration of matrix decomposition - note that in this example, `n=2`:

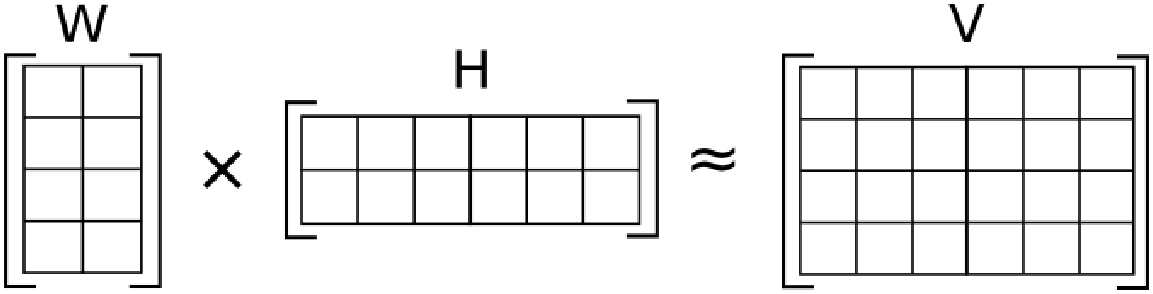

S1 Figure 2. Representation of spatial (W) and temporal (H) components of image time-series dataset V.

Data import

After installing and opening PyAnthem, a dataset in the .mat or .h5 format can be loaded using the **File** dialog (**S1 Figure 1A**). The file should at least include an H (n x t) variable, for audio-only output, or both H (n x t) and W (s_x x s_y x n) variables for audiovisualization. An additional single float variable will be interpreted as the data’s frame rate. Once loaded, video and audio previews are displayed in the bottom and right panels using default settings (**S1 Figure 1D,E**).

Parameter tuning

A variety of video and audio parameters can be changed and previewed using the associated panes in the top left area of the GUI. Parameter adjustments are made in one of the three parameter categories: **File Parameters**, **Video Parameters**, and **Audio Parameters** (**S1 Figure 1B**). The GUI permits selection of the color-scale for visualization including component order, as well as background subtraction and audio digitization settings. The musical scale and instrument to be rendered can also be selected. Changes are reflected in the plots when clicking the **Update** button on the middle left side of the GUI window, and most errors will be indicated in the status box next to the **Update** button (**S1 Figure 1C**). Configurations can be saved and re-loaded for application to multiple datasets.

Spatial components and their dynamic merge can be previewed by using the scroll bar below the **Video** preview plot **S1 Figure 1D**). Audio components can also be previewed using the **Preview** button in the **Audio Parameters** column. The PyAnthem GUI comes with 3 installed instruments to choose from – Grand Piano, Electric Piano, and Strings. PyAnthem can only render audio using one instrument and dataset at a time, but it is possible to merge together multiple separately generated audio streams

using included functions. Data can also be saved in MIDI format for more complex rendering in a 3rd party VST such as Garage Band or REAPER.

File output and merging

Files can be output individually using **Save → Audio** or **Save → Video** (**S1 Figure 1**). These output files can then be merged using **Save → Merge A/V**.

Separately generated audio and video streams can also be merged using command line functions.

Details and installation guide:

Requirements

Python 3.7:

Currently, pyanthem is tested to work on Python 3.7. This will be updated as more versions are tested.

FFmpeg:

ffmpeg enables video creation and merging.

FluidSynth:

FluidSynth enables conversion of MIDI files to crisp, high quality sound files.

If you do have the above requirements installed, you can install pyanthem using pip: `pip install pyanthem`

Conda (optional, but highly recommended):

Conda enables simple and reliable package installation. Use Miniconda for a minimal installation, or Anaconda otherwise.

Installation

Note: If you do not have working installations of the listed requirements (Python 3.7 + fluidsynth + ffmpeg), it is **strongly recommended** that you use Miniconda/Anaconda for a straightforward installation process. If you do not have either, Miniconda is preferred as it is a faster install and takes up much less space than Anaconda.

Using Miniconda/Anaconda:

Create an environment and install the required packages:

```
conda create -n pyanthem python=3.7 pip ffmpeg fluidsynth --channel conda-forge --
channel nicthib
```

Next, activate the environment:

```
conda activate pyanthem
```

Finally, install the pyanthem Python package using pip:

```
pip install pyanthem
```

Example datasets and Scripts

If you want to get familiar with the datasets that pyanthem uses and try some applications of pyanthem, clone this repository or download here.

Using pyanthem in a Jupyter Notebook

To access the pyanthem environment in a Jupyter notebook, first install ipykernel in your environment:

```
conda install -c anaconda ipykernel
```

After this, create the kernel:

```
python -m ipykernel install --user --name=pyanthem
```

Once in a notebook, switch to the pyanthem kernel by selecting `Kernel > Change kernel > pyanthem`
Note: While the pyanthem kernel will now be available in any Jupyter notebook session, pyanthem will not function properly unless the Jupyter notebook is launched inside the pyanthem environment.

Examples

Using pyanthem in GUI mode

First, import pyanthem and begin a pyanthem session:

```
import pyanthem
pyanthem.run()
```

Note: You may run into an error here where some packages are missing. Simply install them using pip, and try running pyanthem again.

The first time you run pyanthem, it will download a necessary soundfont file - this will take a minute or two.

```
♬ Initializing soundfont library...

♬ Downloading 17QuXRbApe0JTlYfBs7iSMCMu3xRWMHOV into...

♬ 238.3 MiB Done.
```

Once completed, the pyanthem GUI will initialize:

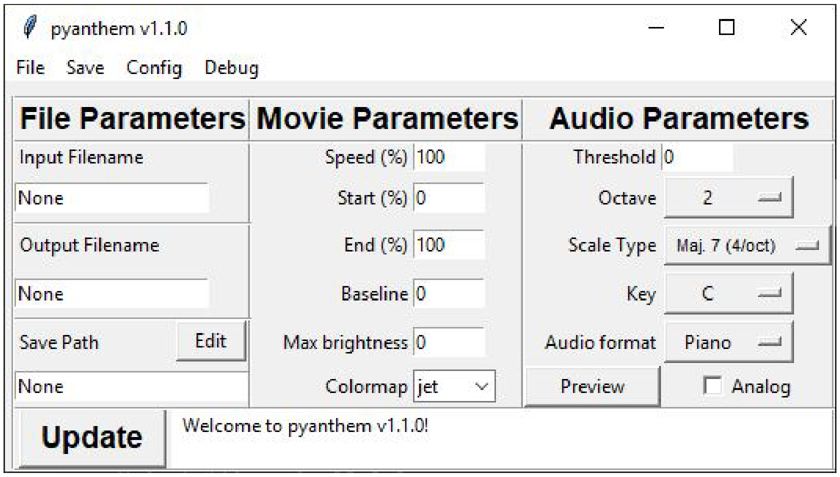

S1 Figure 3. Pyanthem data load interface

Next, load a dataset by clicking `File > Load from .mat`. For this section, we will load the dataset `demo1.mat`. Currently, you can import any .mat or hdf5 file that contains the following variables:

1. Temporal variable (**H, required**): A 2D matrix of shape `[n,t]`, where each row is a component and each column is a time-point. This variable is referred to as **"H"** in the pyanthem environment.
2. Spatial variable (**W, optional**): A 3D matrix of shape `[h,w,n]`, where h and w represent the spatial height and width of your dataset. If this variable is not given, no video output is possible.
3. Framerate (**fr, optional**): A single float value, representing the frame rate of your dataset in Hz. If a framerate is not given, pyanthem will provide a default.

Note: Make sure to only include these variables in your file to avoid any errors. You can name them however you like, but make sure there are only one of each variable.

Once loading is complete, the GUI should update with default options, and plots of **H** and **W**:

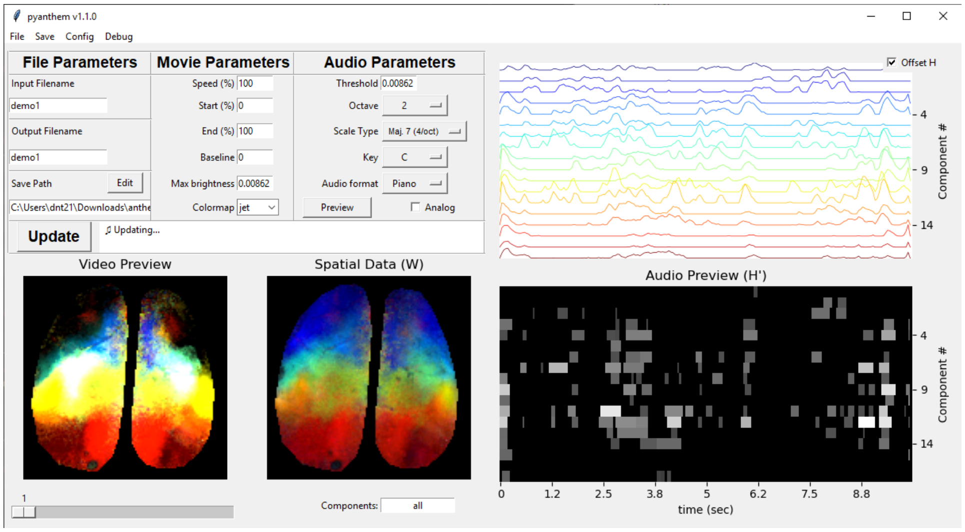

S1 Figure 4. Pyanthem interface after data has been loaded

The bottom left plots show two representations of the dataset: A preview of the output movie (left), and a visualization of what components are included and the colormap selection. The right two plots show raw representations of **H** (top), and a visualization of the audio output file (right). Lighter colors indicate loud notes, and darker colors indicate quiet notes, with black indicating silence.

From here, you can adjust parameters, preview the output, and finally save video and audio files. If you want to check how your parameter adjustments impact your audivisualization, click the **Update** button, and your changes will be reflected. Any issues with your selected parameters will be indicated in the white status box. Try adjusting a few parameters and observing how the plots change.

Finally, render output files with the `Save --> Write A/V then merge` menu command.
Congratulations - you've created your first audiovisualization!

Using pyanthem in CLI (command-line interface) mode

pyanthem's CLI mode is useful for running batch conversions of large amounts of data once you are happy with your audiovisualization parameters, or creating more complex audiovisualizions that use multiple datasets and instruments. CLI mode is not recommended to use until you have used the GUI and are comfortable with the parameters and usage.

To run pyanthem in CLI mode, pass the argument `display=False`, and assign the `.run()` method to a variable:

```
import pyanthem
g=pyanthem.run(display=False)
```

Next, load a dataset and config file using the `.load_data()` and `.load_config()` methods. You can pass an explicit file name to the `file_in` argument, or pass none to recieve a file select prompt (note the use of the leading `r` when naming a file location):

```
g.load_data(file_in=r'path/to/your/file.mat')
g.load_config(file_in=r'path/to/your/config.p')
```

Finally, render the audio and video file, then merge the files using the:
`.write_audio()`, `.write_video()` and `.merge()` methods:

```
g.write_audio()
g.write_video()
g.merge()
```

Once you're comfortable with this syntax, you can combine all of these steps into a single line, write a merged video with the `.write_AV()` method, and even remove the intermediate files using the `.cleanup()` method:

```
data_file = r'path/to/your/file.mat'
config_file = r'path/to/your/config.p'
g.load_data(file_in=data_file).load_config(file_in=config_file).write_AV().cleanup()
```

Congratulations - you've created your first audiovisualization in CLI mode!
