# Supplementary figures and images for: Audiovisualization of real-time neuroimaging data
Source: PLoS One. 2024 Feb 21;19(2):e0297435. doi: 10.1371/journal.pone.0297435 (PMC10881001; doi:10.1371/journal.pone.0297435)

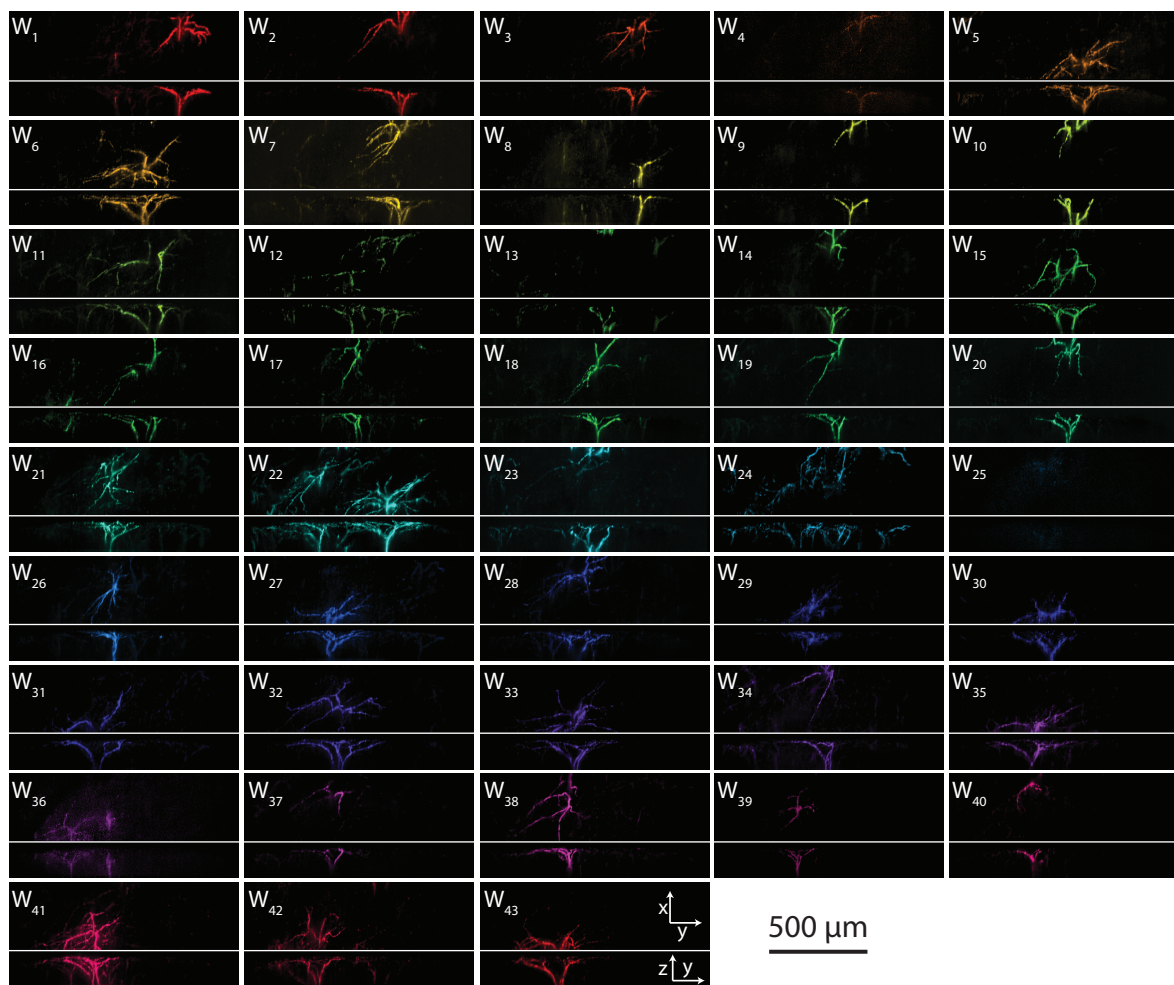

Supplement: S2 Fig — Each panel shows top and side maximum intensity projections (MIPs) of spatial (W) components. (PDF) [file pone.0297435.s003.pdf]
