# Supplementary material for: Audiovisualization of real-time neuroimaging data
Source: PLoS One. 2024 Feb 21;19(2):e0297435. doi: 10.1371/journal.pone.0297435 (PMC10881001; doi:10.1371/journal.pone.0297435)
Supplement: S1 File — (PDF) [file pone.0297435.s008.pdf]

Supplemental Figures

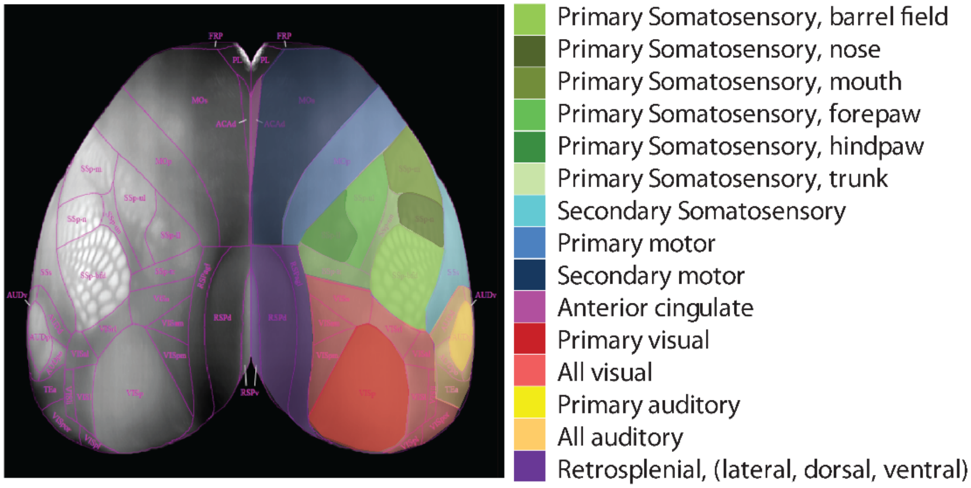

Supplemental Figure 1 - Color-coded cortical functional area atlas for a similar view to that captured in WFOM data. Adapted from the Allen Institute Brain Atlas.

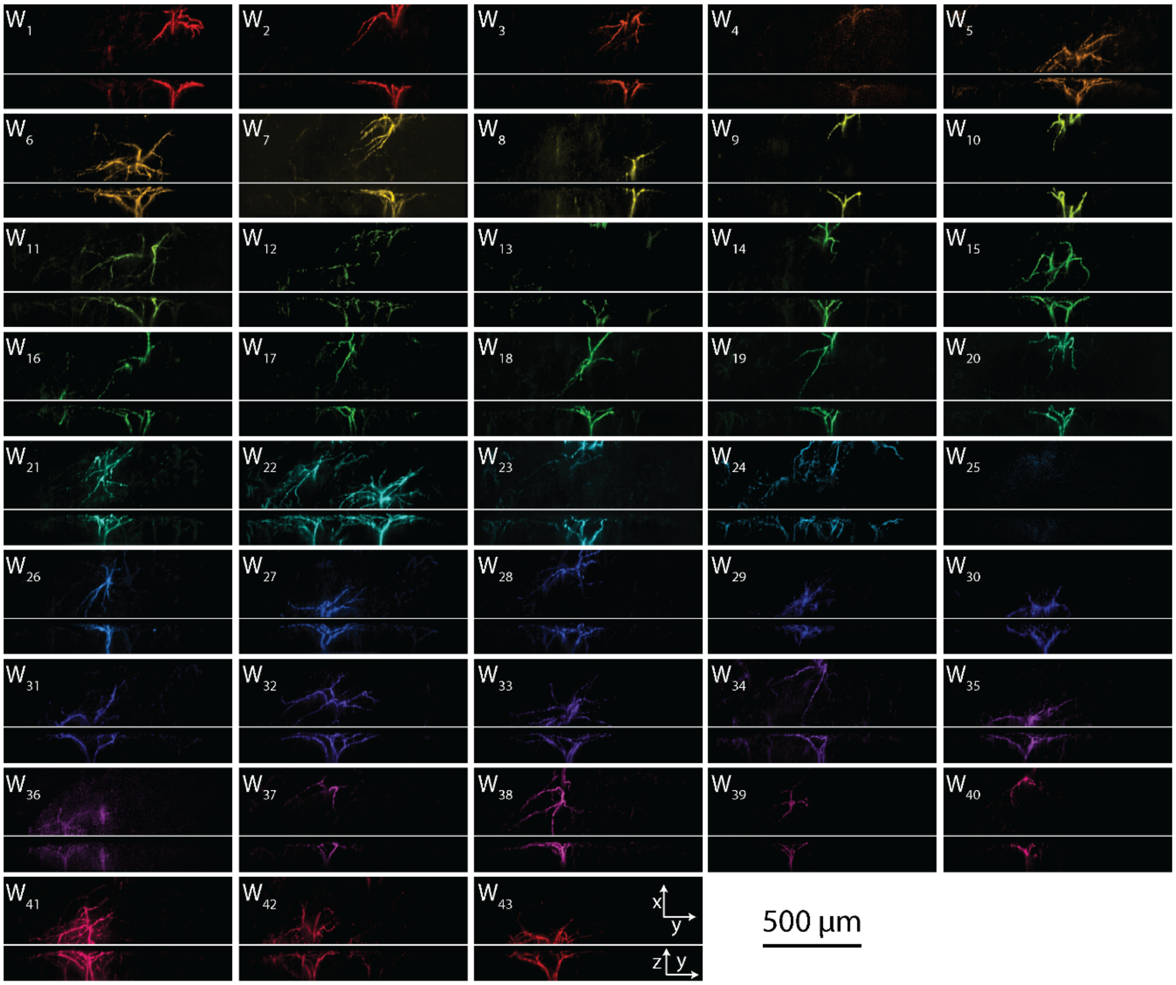

Supplemental Figure 2– All spatial components of dendrites extracted from 4D SCAPE microscopy dataset (experiment 2). Each panel shows top and side maximum intensity projections (MIPs) of spatial (W) components.

Supplemental Movies

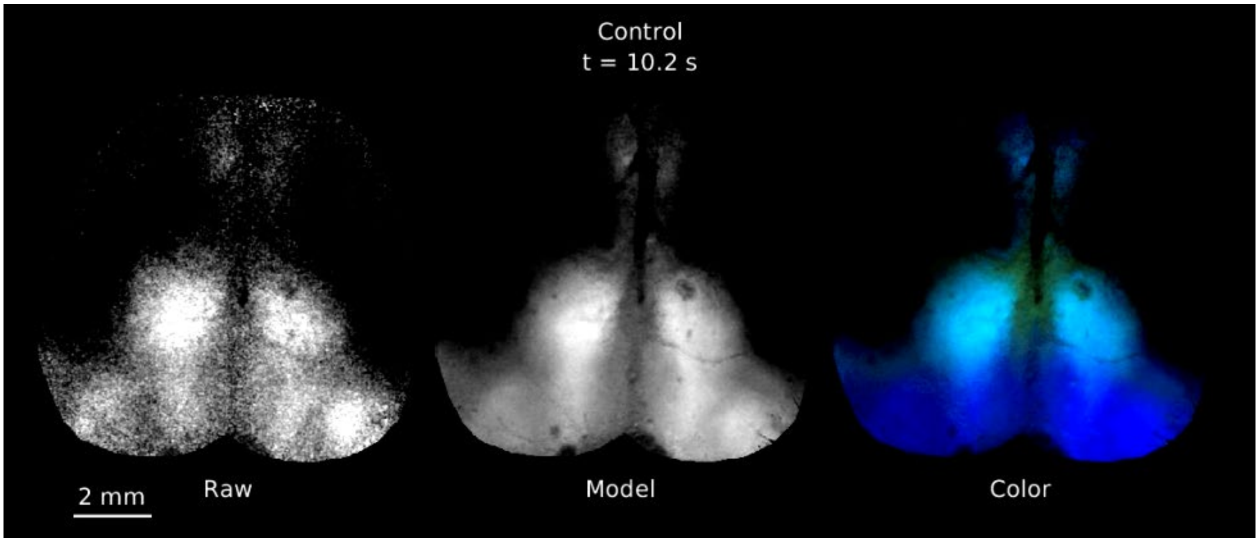

Supplemental Movie S1 – Audiovisualization of Neural activity from the dorsal surface of the thinned skull cortex of the awake mouse. Left: Raw GCaMP activity. Middle: Spatiotemporally unmixed linear model, created by multiplying temporal H (obtained by k-means clustering into 18 ROIs) with spatial W (obtained as an output of NNLS, where H was used as the input). Right: color remixed reconstructed model data, where each component of W was assigned a unique color from the jet color map, arranged from top (red) to bottom (blue). Movie's soundtrack uses analog (sine-wave) based audio encoding of temporal patterns of each spatial component, ordered in an ascending $Cmin^7$ scale from the back (bottom) to the front (top) of the brain. *Note: Movie has sound.*

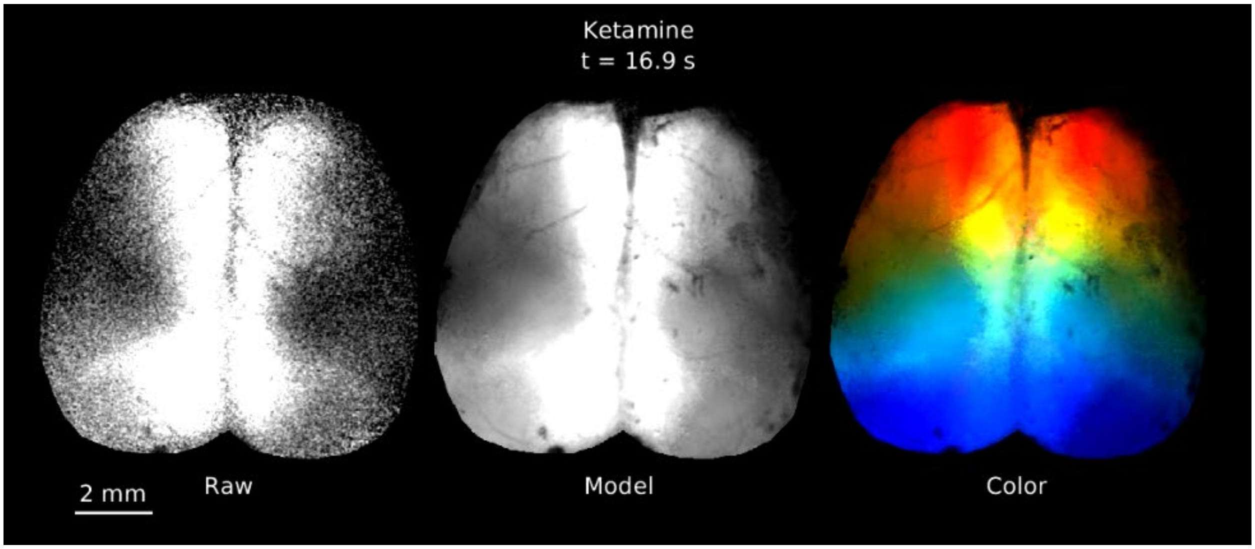

Supplemental Movie S2 – Audiovisualization of Neural activity from the dorsal surface of the thinned skull cortex of the ketamine/xylazine anesthetized mouse. Left: Raw GCaMP activity. Middle: Reconstructed data, created by multiplying temporal H (obtained by k-means clustering into 18 ROIs) with spatial W (obtained as an output of NNLS, where H was used as the input). Right: color remixed reconstructed model data, where each component of W was assigned a unique color from the jet color map, arranged from top (red) to bottom (blue). Movie's soundtrack uses analog (sine-wave) based audio encoding of temporal patterns of each spatial component, ordered in an ascending $Cmin^7$ scale from the back (bottom) to the front (top) of the brain. *Note: Movie has sound.*

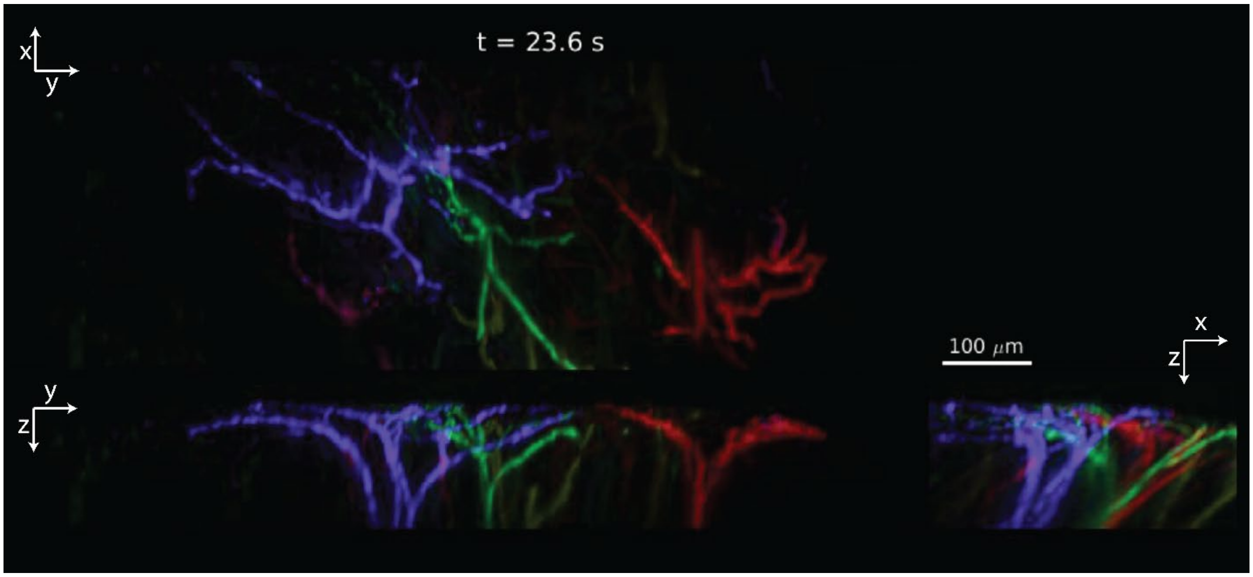

Supplemental Movie S3 – Audiovisualization of SCAPE microscopy data capturing calcium activity in apical dendrites in the awake mouse brain. Panels show top and side maximum intensity projections of color-encoded re-mixed spatial components. Dimensionality reduction was applied to voxels in which at least 5% of values over time exceeded a z-score of 4. Time-course from these voxels were then k-means clustered, and the resulting timecourses were used as an input for NNLS. 43 output components were color-coded from left to right using an HSV color map. Movie's soundtrack depicts supra-threshold events as piano notes and were chosen on an ascending scale according to order of activity. *Note: Movie has sound*.

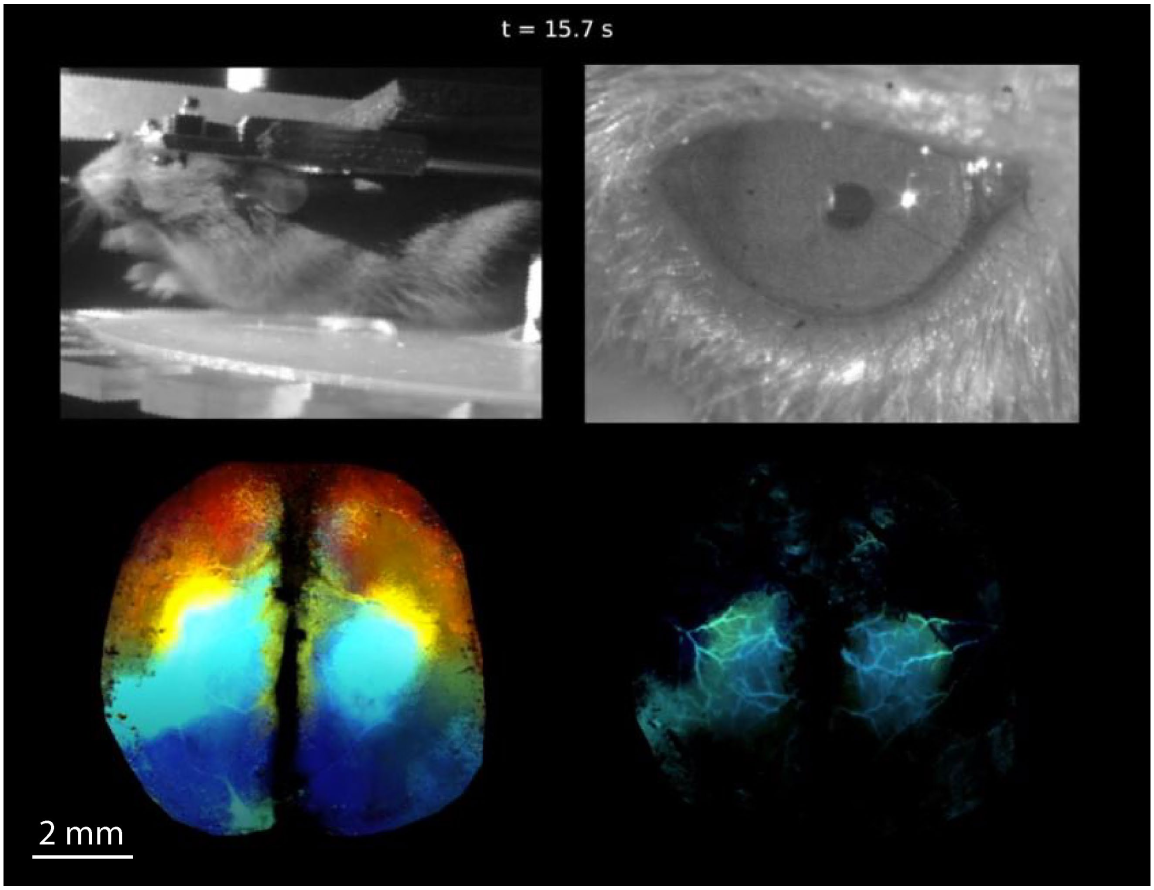

Supplemental Movie S4 – Audiovisualization of neural activity and blood flow from the dorsal surface of the thinned skull cortex of the awake mouse. Behavioral data (top), neuronal activity (GCaMP6f) (bottom left), and cortical hemodynamics (bottom right). Webcam data was acquired simultaneously using two PS3 Eye webcams. Raw GCaMP data was k-means clustered to derive regions of interest (ROIs) from which to extract 12 basis time-courses from both neural and hemodynamic data-streams. Corresponding spatial components fitting a linear model to the original data were derived using non-negative least-squares fitting. Spatial components were then color-coded and re-combined for both datasets, with colors from the Matlab™ jet color map ordered from the front (top, red) to the back (bottom, blue) of the brain. Time-courses for each ROI were converted into audible representations, combined in the movie's soundtrack as piano notes for neural activity and violin as hemodynamics. *Note: Movie has sound*.
