# Supplementary material for: Audiovisualization of real-time neuroimaging data
Source: PLoS One. 2024 Feb 21;19(2):e0297435. doi: 10.1371/journal.pone.0297435 (PMC10881001; doi:10.1371/journal.pone.0297435)
Supplement: S1 Fig — Adapted from the Allen Institute Brain Atlas. (PDF) [file pone.0297435.s002.pdf]

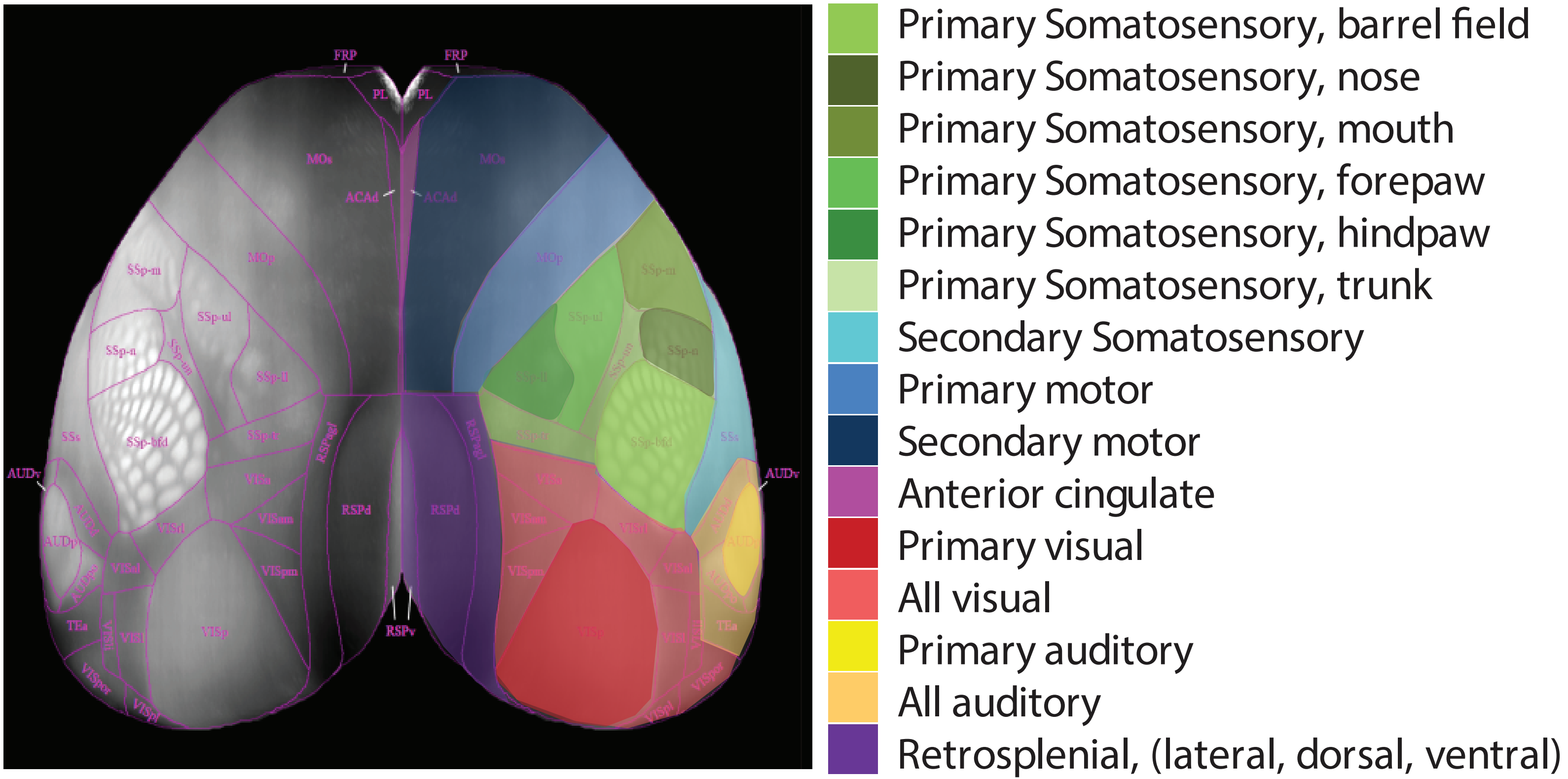
FRP
FRP
PL
PL
MOs
MOs
ACAd
ACAd
MOp
MOp
SSp-m
SSp-m
SSp-ul
SSp-ul
SSp-n
SSp-n
SSp-un
SSp-un
SSp-ll
SSp-ll
SSp-tr
SSp-tr
SSs
SSs
SSp-bfd
SSp-bfd
RSPagl
RSPagl
AUDv
AUDv
AUDd
AUDd
AUDp
AUDp
AUDpo
AUDpo
VISa
VISa
VISam
VISam
VISrl
VISrl
VISal
VISal
VISpm
VISpm
RSPd
RSPd
TEa
TEa
VISli
VISl
VISl
VISp
VISp
RSPv
VISpor
VISpor
VISpl
VISpl
Primary Somatosensory, barrel field
Primary Somatosensory, nose
Primary Somatosensory, mouth
Primary Somatosensory, forepaw
Primary Somatosensory, hindpaw
Primary Somatosensory, trunk
Secondary Somatosensory
Primary motor
Secondary motor
Anterior cingulate
Primary visual
All visual
Primary auditory
All auditory
Retrosplenial, (lateral, dorsal, ventral)
